# The Comet Assay and its applications in the field of ecotoxicology: a mature tool that continues to expand its perspectives

**DOI:** 10.3389/fgene.2015.00180

**Published:** 2015-06-04

**Authors:** Joaquín de Lapuente, Joana Lourenço, Sónia A. Mendo, Miquel Borràs, Marta G. Martins, Pedro M. Costa, Mário Pacheco

**Affiliations:** ^1^Unit of Experimental Toxicology and Ecotoxicology (UTOX-CERETOX), Barcelona Science ParkBarcelona, Spain; ^2^Department of Biology & CESAM, University of Aveiro, Campus Universitário de Santiago. Aveiro, Portugal; ^3^Departamento de Ciências e Engenharia do Ambiente, MARE – Marine and Environmental Sciences Centre, Faculdade de Ciências e Tecnologia da Universidade Nova de LisboaCaparica, Portugal

**Keywords:** Comet Assay, ecotoxicology, piscine model, amphibians, earthworms, mollusks, plants

## Abstract

Since Singh and colleagues, in 1988, launched to the scientific community the alkaline Single Cell Gel Electrophoresis (SCGE) protocol, or Comet Assay, its uses and applications has been increasing. The thematic areas of its current employment in the evaluation of genetic toxicity are vast, either *in vitro* or *in vivo*, both in the laboratory and in the environment, terrestrial or aquatic. It has been applied to a wide range of experimental models: bacteria, fungi, cells culture, arthropods, fishes, amphibians, reptiles, mammals, and humans. This document is intended to be a comprehensive review of what has been published to date on the field of ecotoxicology, aiming at the following main aspects: (i) to show the most relevant experimental models used as bioindicators both in the laboratory and in the field. Fishes are clearly the most adopted group, reflecting their popularity as bioindicator models, as well as a primary concern over the aquatic environment health. Amphibians are among the most sensitive organisms to environmental changes, mainly due to an early aquatic-dependent development stage and a highly permeable skin. Moreover, in the terrestrial approach, earthworms, plants or mammalians are excellent organisms to be used as experimental models for genotoxic evaluation of pollutants, complex mix of pollutants and chemicals, in both laboratory and natural environment. (ii) To review the development and modifications of the protocols used and the cell types (or tissues) used. The most recent developments concern the adoption of the enzyme linked assay (digestion with lesion-specific repair endonucleases) and prediction of the ability to repair of oxidative DNA damage, which is becoming a widespread approach, albeit challenging. For practical/technical reasons, blood is the most common choice but tissues/cells like gills, sperm cells, early larval stages, coelomocytes, liver or kidney have been also used. (iii) To highlight correlations with other biomarkers. (iv) To build a constructive criticism and summarize the needs for protocol improvements for future test applications within the field of ecotoxicology. The Comet Assay is still developing and its potential is yet underexploited in experimental models, mesocosmos or natural ecosystems.

## Introduction

The extraordinary growth in the chemical industry during the second half of the twentieth century has led to the appearance in nature of thousands of new products every year, a large percentage of which have significant biological effects. The presence in the environment of xenobiotics that are biologically active and difficult to break down represents a degree of stress that is frequently unacceptable for living organisms and that is also expressed at the ecosystem level. Both direct and indirect toxic activity can, in certain circumstances, be an important risk factor for the human population as well.

The usual way to approach ecotoxicity testing, according to relevant EPA and OECD guidelines for the testing of chemicals (for example, in the context of REACH normative) or of veterinary drugs, is the use of well-defined tests, in which an array of selected species, representing the main trophic levels, are exposed to a single pollutant under controlled laboratory conditions. Such a standardized approach is necessary to acquire information in a relatively short time, to gather data easy to compare and to interpret and, of course, for regulatory purposes. However, extrapolation to real world is challenging if at all feasible.

Models to study environmental toxicity are a necessary compromise between the control of experimental parameters (through the use of lab-reared substitute species and the setting of a thoroughly controlled exposure scenario) and realism (field or semi-field studies). An entirely different approach is based on the use of native species, which essentially considers pollution as a complex situation and therefore implies a more holistic interpretation of the real conditions of exposure in the field. This kind of study includes the capture of animals and/or the collection of plants, water or soil samples on the field. This approach allows considering interactions among pollutants and also homeostasis. Life-term exposure occurs in a natural context, allowing the action of such modulating factors as discontinuous pattern of pollution, reduction of the animal activity or sheltering. Interpretation of the results, on the other hand, may be particularly difficult in face of the many constraints and confounding factors of the natural environment (Borràs and Nadal, 2004).

The term *mutagen* refers to a substance that induces transmissible changes in DNA structure (Maurici et al., 2005), involving a single gene or a group of genes. Genotoxins are a broader category of substances which induce changes to the structure or number of genes via chemical interaction with DNA and/or non-DNA targets (Maurici et al., 2005). The term *genotoxicity* is generally used unless a specific assay for mutations is being discussed. A large number of assay systems have been established for the measurement of genetic toxicity of chemical and physical agents. The Comet Assay, or Single Cell Gel Electrophoresis (SCGE), is a standard method for determining *in vivo*/*in vitro* genotoxicity. It offers a simple way of evaluating the damage caused by a clastogenic agent by measuring breaks in the DNA chain of animal and plant cells. One of the most striking features of the Comet Assay is the versatility, which allows its application to a wide array of different cell types and matrices. This characteristic, as well as its sensitivity, makes it especially well-suited for ecotoxicological studies, both in the terrestrial and the aquatic compartment.

Although, for different reasons, water has been a privileged scenario for the pioneering studies on environmental genotoxicity, soil remains the primary way of entry into the environment for a number of pollutants, going from agricultural pesticides to veterinary drugs. As a consequence, testing species representative of the trophic chain in both compartments is relevant and necessary to thoroughly assess the genotoxic effects of environmental pollutants. In either case, it is clear that in the last decades the Comet Assay has been applied to a wide range of scenarios, species and ecogenotoxicity assessment approaches. As such, the present paper primarily aims to critically reviewing the application and technical developments of this versatile protocol in the context of ecotoxicology.

## Experimental models

### Amphibians

Amphibians are among the most sensitive organisms to environmental changes, mainly due to an early aquatic-dependent development stage and a highly permeable skin. As such, they have been proposed as bioindicators of environmental contamination (Gonzalez-Mille et al., 2013). Environmental contaminants are pointed out as the primary cause in the decline of amphibian populations, hence the importance of evaluating exposure and sublethal effects in amphibian monitoring programs (Gonzalez-Mille et al., 2013). Nonetheless, the application of the Comet Assay in ecotoxicological studies involving these organisms is relatively new. The first work reported dates from 1996 (Ralph et al., 1996). Since then, a number of studies have been conducted that apply the Comet Assay to amphibian cells in adult and larval stages of several species, mainly *Lithobates clamitans* and *Xenopus laevis*. These studies focused mainly on the determination of the exposure effects to several contaminants, such as, for instance: herbicides (Clements et al., 1997; Liu et al., 2006, 2011; Yin et al., 2008; Meza-Joya et al., 2013), pesticides (Feng et al., 2004; Yin et al., 2009; Ismail et al., 2014) and other xenobiotics as methyl methanesulfonate (Ralph et al., 1996; Ralph and Petras, 1998b; Mouchet et al., 2005a). Reports on the effects of the exposure to fungicides (Mouchet et al., 2006a), metals (Wang and Jia, 2009; Zhang et al., 2012), petrochemical contaminants (Huang et al., 2007), Persistent Organic Pollutants (POPs) (Gonzalez-Mille et al., 2013), ethyl methanesulfonate (Mouchet et al., 2005a); benzo(a)pyrene (Mouchet et al., 2005a), sulfur dyes (Rajaguru et al., 2001), antibiotics (Banner et al., 2007; Valencia et al., 2011), and dimethyl sulfoxide (DMSO) (Valencia et al., 2011) may also be found. Additionally, the biomonitoring of contaminated sites recurring to the Comet Assay in amphibians has also been performed, namely, of chemically-polluted lakes (Erismis et al., 2013), coal mines (Zocche et al., 2013), waste dumping sites (Maselli et al., 2010), dredged sediments (Mouchet et al., 2005b), polluted water bodies (Ralph and Petras, 1997, 1998a) and residues from municipal solid waste incineration (Mouchet et al., 2006b). Studies have also been reported where on sperm cells (Shishova et al., 2013) and the effects of exposure to electromagnetic fields (Chemeris et al., 2004) were assessed by the Comet Assay. Generally, studies are conducted *in vivo* and erythrocytes are the cell type most commonly used.

### Piscine models

Historically, fishes are closely linked with the transposition of the Comet Assay to the field of environmental toxicology, since they are among the first animal models to which the technique was adopted as a biomonitoring tool to assess the genotoxicity of contaminants on wildlife. A pioneering application was carried out by Pandrangi et al. (1995). This study examined the effects of toxic wastes accumulated in the sediment of the Great Lakes (Canada) and the sentinel species selected were the brown bullhead (*Ameiurus nebulosus*) and the common carp (*Cyprinus carpio*). The alkaline procedure developed and reported by Singh et al. (1988) was successfully adapted to fish erythrocytes, albeit the introduction of a few modifications. The authors concluded that the assay “is extremely sensitive and should be useful in detecting DNA damage caused by environmental contaminants.” Since 1995, this premonitory statement has been recurrent and increasingly reinforced by an array of scientific publications, exploring a wide diversity of approaches, viz. *in vitro* (Kienzler et al., 2012), *ex vivo* (Santos et al., 2013), *in vivo* (Palanikumar et al., 2013), and *in situ* (Srut et al., 2010) exposures, as well as surveying wild native specimens (Laroche et al., 2013).

To date, more than 300 articles have been published addressing DNA integrity in fish cells through the Comet Assay, making fish by far the most adopted animal group in the framework of environment health assessment. Furthermore, in recent years we have witnessed to an even greater profusion of publications. In 2013, for instance, 43 scientific articles were published (according to a literature search on PubMed) evaluating DNA damage by Comet Assay in piscine models (including fish cell lines) exposed to various potentially genotoxic agents. This vast utilization of fish should also be regarded as reflecting a primary concern of genetic ecotoxicologists over the health status of aquatic ecosystems. As a further evidence of the Comet Assay popularity as a tool for detecting DNA strand breaks in fish (along with other aquatic animals) it should be underlined that this subject has been periodically reviewed in 1998 (Mitchelmore and Chipman, 1998), 2003 (Lee and Steinert, 2003), and 2009 (Frenzilli et al., 2009).

It is well-established that Comet Assay is applicable, virtually, to all species. A clear demonstration of this polyvalence is the finding that, since 1995, this assay was successfully adapted to more than 90 fish species. This wide range of species includes mostly bony fish (Class Osteichthyes), both ray-finned fishes (Subclass Actinopterygii), the overwhelming majority of cases, and lobe-finned fishes (Subclass Sarcopterygii) like *Arapaima gigas* (Groff et al., 2010). The jawless fish (Class Agnatha) are represented with an interesting study with sea lamprey (*Petromyzon marinus*) describing the relationship between sperm DNA damage and fertilizing ability (Ciereszko et al., 2005), while cartilaginous fish (Class Chondrichthyes) are completely unexplored. Bearing in mind that the Comet protocol requires very small cell samples, the technique showed to be suitable for a broad variety of fish sizes, from very small fish (e.g., the mosquitofish *Gambusia holbrooki*; Ternjej et al., 2010), and even fingerlings (e.g., milkfish *Chanos chanos*; Palanikumar et al., 2013), up to bigger species like conger (*Conger conger*; Della Torre et al., 2010).

In what concerns to the type of agent/contaminant tested, the application of Comet Assay in the field of aquatic genotoxicology has accompanied the evolution of other subareas of environmental toxicology involving piscine models. Hence, besides the contaminants traditionally evaluated like POPs (González-Mille et al., 2010), metals (Velma and Tchounwou, 2013), or pesticides (Guilherme et al., 2010), genotoxicologists have shown to be aware to emergent genotoxicants such as pharmaceutical substances (Rocco et al., 2010), endocrine disruptors (e.g., tetrabrombisphenol A; Linhartova et al., 2014), nanoparticles (Taju et al., 2014), biotoxins (Silva de Assis et al., 2013), radionuclides (Stiazhkina et al., 2012), or ultraviolet (UV) radiation (Mekkawy et al., 2010).

### Bivalves and other molluscs

In recent years, the application of the Comet Assay in molluscs has been springing up. These organisms have long been regarded as prime subjects in biomonitoring programmes worldwide, especially, albeit not exclusively, in aquatic ecosystems. Bivalves, in particular, receive special attention both as sentinel and toxicity-testing subjects and a large array of literature has been published in the last few years. Among these, mussels (*Mytilus* spp.) have become one the most important targets when researching on marine genotoxicants using the Comet Assay (in large part owing to their worldwide distribution and known sensitivity to pollutants), from substance testing to the monitoring of sediments and waters *in situ* and *ex situ* and even recovery assessment following oil spills (Thomas et al., 2007; Almeida et al., 2011; Fernández-Tajes et al., 2011; Pereira et al., 2011; Martins et al., 2012, 2013; Dallas et al., 2013). Research on the genotoxic effects of emerging pollutants, including nanomaterials is also arising (Gomes et al., 2013). Other bivalves, of more local relevance, have been shown to be good candidates, such as the clam *Ruditapes decussatus* in SW Europe (Martins et al., 2013) and the cockle *Cerastoderma edule* (Pereira et al., 2011). In freshwater environments, the green-lipped mussel (*Perna* spp.), the zebra mussel *Dreissena polymorpha* and the Asian clam *Corbicula fluminea* are the most common bivalves in genotoxicity assessment through the Comet Assay (Michel and Vincent-Hubert, 2012; Parolini and Binelli, 2012; Chandurvelan et al., 2013; Michel et al., 2013; dos Santos and Martinez, 2014). Gastropods take the place of bivalves in terrestrial environments and the use of snails (like *Helix* spp.) as effective sentinels for genotoxicants has been demonstrated *in situ* (Angeletti et al., 2013).

### Terrestrial organisms

The fate and effects of pollutants on living organisms may differ in the two compartments. Soils are complex associations with high binding capacity to both inorganic and organic molecules, which may, as well as certain modifications along time (e.g., aging and weathering), modulate the biological effects of contamination. For these reasons, toxicity to terrestrial species cannot be directly extrapolated from aquatic species, meaning that specific approaches and models are needed to assess the impact of soil pollutants on terrestrial biota (Vasseur and Bonnard, 2014).

The role that filtering organisms, like mussels, play in water is covered in soil by earthworms, which, in addition, are able to move around and prospect its surroundings, giving information both on the temporal (accumulation) and the spatial axis. Plants, in turn, are sessile, but expand their roots both laterally and in depth, absorbing pollutants from successive strata.

The application of Comet Assay to earthworms, and consequently the use of such extraordinary prospectors as sentinels for the presence of genotoxicants in soil, started in the nineties of the last century (Singh et al., 1988; Verschaeve and Gilles, 1995; Salagovic et al., 1996), and since then has been extensively revised (Cotelle and Férard, 1999; Espinosa-Reyes et al., 2010; Liu et al., 2010; Atli Şekeroglu et al., 2011; Lionetto et al., 2012; Andem et al., 2013; Vernile et al., 2013; Fujita et al., 2014; Vasseur and Bonnard, 2014; Zhang et al., 2014). Several earthworms comparative studies have been performed (Vasseur and Bonnard, 2014). *Eisenia fetida* and *Aporrectodea caliginosa* showed an equivalent sensitivity, as assessed by Comet Assay (Klobučar et al., 2011). Fourie et al. (2007) compared the sensitivity of five earthworm species (*Amynthas diffringens, A. caliginosa, E. fetida, Dendrodrilus rubidus* and *Microchaetus benhami*) to Cd genotoxicity after a 48 h-exposure. *E. fetida* presented the highest percent of DNA in tail and was the second most sensitive species after *D. rubidus*, which showed the highest increase in DNA breaks compared with the control.

Plants are also specially well-fitted for ecotoxicological assessment of soils, including genotoxicity. The Comet Assay may be performed in different organs (nucleus of roots cells or leaf cells), and combined, when suitable, with growth tests (Grant, 1994; Sandhu et al., 1994; Gopalan, 1999; Ma, 1999; Sadowska et al., 2001; Ma et al., 2005). However, cell lysis and release from plant cells is challenging and require special adaptations to the protocol (such as mechanical extraction of nuclei or protoplast production), which may be tissue– and species–dependent (see Costa et al., 2012a and references therein). In general, the Comet Assay in plants is far from being as common and widespread as in animals.

Genotoxicants in the terrestrial compartment have also been tracked by means of Comet Assay using vertebrates as sentinel species, particularly avian and rodents. The ecological disaster occurred in April 1998 in the mines of Aznalcóllar, consisting in a massive toxic spill of acid waste containing metals, threatened the wildlife in the Doñana National Park in SW Spain. The presence of DNA damage was studied along 4 years by means of Comet Assay in white storks (*Ciconia ciconia*) and black kites (*Milvus migrans*) (Pastor et al., 2001, 2004; Baos et al., 2006). Results indicate that the exposed birds had a significantly increased level of genotoxic damage compared with control animals from non-contaminated locations, that the toxic spill still appears to be affecting the wildlife 4 years after the mining disaster and that attempts at cleaning up the waste have proved ineffective based on DNA damage detection. A study to determine DNA damage in blood cells of barn swallows (*Hirundo rustica*) inhabiting the Chernobyl region was carried out, to evaluate whether chronic exposure to low-level radioactive contamination continues to induce genetic damage in free-living populations of animals. The results showed that Comet values in barn swallows living in areas surrounding Chernobyl are still increased when compared to swallows sampled at low-level sites, even 20 years after the accident at the Chernobyl nuclear power plant (Bonisoli-Alquati et al., 2010).

Rodent species have been used as sentinels of eco-genotoxicity in a variety of scenarios. The European wood mice (*Apodemus sylvaticus*) is a ubiquitous, abundant species which has been studied to assess the effects of dumping sites (Delgado et al., 2000), urban or traffic pollution (Borràs and Nadal, 2004) or the surroundings of an abandoned uranium mining site (Lourenço et al., 2013). In all these cases, the combination of Comet Assay and wood mice proved to be a sensitive and reliable tool for the detection of the exposure to environmental genotoxicants. The yellow-necked wood mouse (*Apodemus flavicollis*) is a closely–related species inhabiting the regions of central and northern Europe. A study was performed in different protected areas of the Strandzha National Park in Bulgaria in 2010 and 2011. An increase in the Comet Assay parameters in the analyzed individuals of yellow-necked mouse from the Sredoka protected area was established. Those results indicated that there was genetic damage in some mice populations as a consequence of chronic contamination (Mitkovska et al., 2012). The Algerian mouse (*Mus spretus*) is a similar species, more frequent in south-Europe. This species has been used in different studies, however. A comparison was done between mice living in an industrial area in the neighborhood of Huelva city, SW Spain, and in a natural area (Doñana National Park). Results suggest that Comet Assay in wild mice can be used as a valuable tool in pollution monitoring (Mateos et al., 2008). Genotoxicity monitoring using the Comet Assay on peripheral blood leukocytes of the Algerian mouse was carried out in Doñana Park (Spain), after the environmental disaster of the Aznalcollar pyrite mine in 1998. The mice were sampled in different areas 6 months after the ecological disaster and again 1 year later. Results showed that in 1998 Comet parameters were increased in all the areas examined, whereas a significant decrease in the values was observed in the 1999 samples, which were collected in a riverside area subject to tide flows (Festa et al., 2003).

Wild individuals of *Rattus rattus* and *Mus musculus* have also been assessed for DNA damage by the Comet Assay. A study was conducted in a coal mining area of the Municipio de Puerto Libertador, Colombia. Animals from two areas in the coal mining zone and a control area were investigated. The results showed evidence that exposure to coal results in elevated primary DNA lesions in blood cells of rodents (León et al., 2007). Meadow voles (*Microtus pennsylvanicus*) have been used to measure the effects of pesticide exposure in golf courses of the Ottawa/Gatineau region of Canada (Knopper et al., 2005). *Ctenomys torquatus* is a South-American species which was used for biomonitoring in the coal region of Rio Grande do Sul (Brazil). The results of this Comet Assay study indicate that coal and by-products not only induce DNA damage in blood cells, but also in other tissues, mainly liver, kidney, and lung (da Silva et al., 2000a,b).

It is also worth to note how a multi-trophic level approach may be applied to assess the impact of toxicity on a given ecosystem. A recent example is the assessment of the effect of radioactive materials released in 2011 during the accident at Japan's Fukushima nuclear power plant on wildlife. The effects of exposure to environmental radiation were studied by means of Comet Assay in wild boars (*Sus scrofa leucomystax*) and earthworms (*Megascolecidae*). Regions with low (0.28 μSv/h) and high (2.85 μSv/h) levels of atmospheric radiation were compared. The authors constructed a model food web featuring the wild boar as the top predator, and measured the radioactivity levels in soil, plant material, earthworms, and wild boar. The extent of DNA damage in wild boars did not differ significantly between animals captured in the two regions, but earthworms from the “high-dose” region had a significantly greater extent of DNA damage than did those from the “low-dose” region (Fujita et al., 2014).

## A methodological overview

### Amphibians

Over the years, the Comet Assay protocol has undergone some alterations; however there is no clear evolution or tendency (see Table 1). Regarding the lysis buffer, in the first papers published by Ralph et al. (1996) and Ralph and Petras (1998b) and also by Clements et al. (1997) no detergent (e.g., Triton X-100) nor DMSO were added to the stock solution. Later, in 1997 and 1998, Ralph and Petras (1997, 1998a), added these components to the lysis buffer, which made it very similar to the buffers commonly used nowadays in most of the studies published. Ever since, in most of the studies, the buffer includes these two components, with few exceptions (Chemeris et al., 2004; Valencia et al., 2011; Zhang et al., 2012; Meza-Joya et al., 2013). Additionally some variations are also found in the composition of the lysis buffers, such as the inclusion or exclusion of some commonly used reagents like, for example, the replacement of sodium sarcosinate with SDS as detergent. However, in two particular studies performed by Valencia et al. (2011) and also Meza-Joya et al. (2013), a different lysis buffer and lysis protocol is used. These authors exposed the cells to a lysing solution containing proteinase K and calcium chloride, before the cells were mixed with the agarose and spread out on slides. This protocol was used in blood cells from *Eleutherodactylus johnstonei* to overcome the problem of lysing those cells, which were seemingly resistant to the lysis treatments commonly performed. Thus, this appears to be an important factor to consider in future studies with similar species. Regarding lysis itself, it is usually performed under alkaline conditions, using time intervals varying from 25 min to a maximum of 1 week. Until 2005, lysis was usually performed at room temperature, however, from 2006 until now it is generally conducted at 4°C, which is in agreement to the guidelines published by Azqueta and Collins (2013). The low melting point agarose concentration it is usually 0.5%, but it varies from 0.4 to <1%, which limits the comparison of the results obtained in the various studies, since it directly affects DNA migration. Accordingly, the higher the agarose concentration, the lower the % tail DNA (Azqueta and Collins, 2013). Denaturation is generally conducted in alkaline conditions (pH > 13), from 5 min to 40 min which, once again, limits the comparison between studies, since it also affects DNA migration. As referred by Azqueta and Collins (2013), the higher the incubation period the higher the % tail DNA. Regarding electrophoresis, voltage can vary between 18 and 27 V, generally at 300 mA, from 4 to 50 min. However, not all the studies refer the voltage gradient used (V/cm), and therefore a comparison between studies is still a limitation. Generally, variation between protocols, mainly regarding agarose concentration, denaturation and electrophoresis conditions, denotes lack of standardization, compromising direct comparisons between studies.

**Table 1:** **Summary of the methodological developments on amphibians**.

**Experimental model**	**Contaminants tested**	**Cell type/tissue**	**Agarose (%)**	**Lysis buffer composition**	**Lysis conditions**			**Denaturation conditions**		**Electroforesis conditions**		**References**
					**pH**	**Temp. (°C)**	**Duration (h)**	**pH**	**Duration**	**Voltage**	**Duration**	
*Lithobates clamitans* *Anaxyrus* *americanus* *Lithobates* *catesbeianus* (tadpoles)	MMS Atrazine Metalochlor Glyphosate Metribuzin 2,4-D amine	Blood	0.4 0.4 0.5	Buffer A: 2.5 M NaCI, 100 mM Na_2_EDTA, 10 mM Tris, 10% DMSO, 1% Na - sarcosinate	10	RT	2	13	15 min	25 V (265–270 mA)	20 min (37°C)	Ralph et al., 1996; Clements et al., 1997; Ralph and Petras, 1998b
*Anaxyrus* *americanus* *Lithobates clamitans* *Lithobates pipiens* (tadpoles)	Several pollutants	Blood	0.4	Buffer B: 2.5 M NaCl, 100 mM Na_2_EDTA, 10 mM Tris, 10% DMSO, 1% Na - sarcosinate, 1% Triton X-100	10	RT	2	13	15 min	25 V (265–270 mA)	20 min (37°C)	Ralph and Petras, 1997, 1998a
*Euphlyctis hexadactylus* (tadpoles)	Sulfonated dyes	Blood	0.75	Buffer C: 2.5 M NaCl, 100 mM Na_2_EDTA, 10 mM Tris, 10% DMSO, 1% Triton X-100	10	RT	2	13	25 min	20 V (300 mA)	20 min	Rajaguru et al., 2001
*Xenopus laevis* (adults)	High peak-power pulsed electromagnetic field	Blood	0.5	Buffer D: 2.5 M NaCl, 10 mM Tris, 1% Triton X-100, 100 mM Na_2_EDTA	10	22	25 min	13	20 min	1.9 V/cm	20 min (4°C)	Chemeris et al., 2004
*Pelophylax nigromaculatus* (adults)	Imidacloprid RH-5849	Blood	0.5	Buffer B	10	RT	1	13	25 min	25 V (150 mA)	50 min	Feng et al., 2004
*Xenopus laevis* *Pleurodeles waltl* (tadpoles)	Several pollutants Benzo(*a*)pyrene EMS MMS Captan	Blood	0.5	Buffer C	10	RT	1	13	20 min (4°C)	20V (300 mA)	20 min (Xenopus) 30 min (*Pleurodeles*) (4°C)	Mouchet et al., 2005a,b, 2006a,b
*Strauchbufo raddei* (tadpoles and adults)	Acetochlor Petrochemical contaminants	Blood Hepatocytes	0.6 0.5	Buffer B	10	4	1	13	20 min (4°C)	25 V (300 mA)	20 min (4°C)	Liu et al., 2006; Huang et al., 2007
*Xenopus laevis* *Xenopus tropicalis* (adults)	Bleomycin	Splenic lymphocytes	nm	Buffer B	10	4	30 min	10	30 min (RT)	24 V	4 min (TBE; RT)	Banner et al., 2007
					**pH**	**Temp. (°C)**	**Duration (h)**	**pH**	**Duration**	**Voltage**	**Duration**	
*Bufo gargarizans* (tadpoles)	Acetochlor, Butachlor, Chlorimuron-ethyl Paraquat Chlorpyrifos	Blood Hepatocytes	<0.8	Buffer B	10	4	2	13	30 min (4°C)	18 V (300 mA)	20 min (4°C)	Yin et al., 2008, 2009
*Pelophylax nigromaculatus* (adults)	Lead	Testicular cells	0.3	Buffer E: 2.5 M NaCl, 10mM Na_2_EDTA, 10 mM Tris, 10% DMSO, 1% SDS, 1% Triton X-100, pH 10	10	4	2–4	10	30 min (4°C)	22 V (220 mA)	30 min (4°C)	Wang and Jia, 2009
*Pelophylax lessonae* (adults)	Several pollutants	Blood	0.5	Buffer C	10	4	1	13	nm	20 V (300 mA)	20 min (4°C)	Maselli et al., 2010
*Fejervarya limnocharis* (tadpoles)	Butachlor	Blood	0.9	Buffer B	10	RT	1	13	20 min (RT)	23 V (300 mA)	25 min (RT)	Liu et al., 2011
*Eleutherodactylus johnstonei* (adults)	Bleomycin 4-nitro- quinoline-1-oxide Glyphosate	Blood	0.7	Buffer F: 50 mM Tris, 10 mM CaCl2, 0.04 g/mL proteinase K	8	6 ± 2	10 min	13	25 min	25 V (300 mA, 0.90 V/cm)	30 min (6 ± 2°C)	Valencia et al., 2011; Meza-Joya et al., 2013
*Fejervarya limnocharis* (adults)	Cadmium	Testicular cells	<1	Buffer A	10	RT	2	13	30 min (4°C)	22 V (220 mA)	30 min (4°C)	Zhang et al., 2012
*Hypsiboas faber* (adults)	Heavy metal pollution	Blood	0.7	Buffer C	10	4	1	13	20 min	25 V (300 mA, 0.90 V/cm)	20 min	Zocche et al., 2013
*Rana temporaria* (adults)	–	Sperm cells	0.5	Buffer B	10	4	1	13	20 min	27 V (260–270 mA, 0.2 V/mm)	? (4°C)	Shishova et al., 2013
*Rhinella marina* (adults)	POPs	Blood	0.5	Buffer C	13	4	1 week	13	5 min	25 V (300 mA)	10 min	Gonzalez-Mille et al., 2013
*Pelophylax ridibundus* (adults)	Several pollutants	Blood	0.5	Buffer B	10	4	1	13	40 min	25 V (300 mA)	25 min	Erismis et al., 2013
*Duttaphrynus stomaticus* (adults)	Chlorpyrifos	Blood	0.5	Buffer C	10	4	2	13	25 min	2 5V (300 mA, 0.73 V/cm)	25 min	Ismail et al., 2014

*RT, room temperature*.

### Piscine models

The wide variety of fish species addressed, tissues sampled, and experimental approaches adopted have led to a profusion of adaptations to the Comet Assay protocol (see Table 2). To date, no standardized Comet Assay procedures exist for environmental studies involving fish. In addition, a standardization of sampling protocols when using laboratory exposed or both transplanted and wild specimens in biomonitoring studies is required (Frenzilli et al., 2009).

**Table 2:** **Summary of the methodological developments on piscine models**.

**Experimental model**	**Contaminants tested**	**Cell type/tissue**	**Agarose (%)**	**Lysis buffer composition**	**Lysis conditions**			**Denaturation conditions**		**Electroforesis conditions**		**References**
					**pH**	**Temp (°C)**	**Duration (h)**	**pH**	**Duration**	**Voltage**	**Duration**	
*Ameiurus nebulosus* and *Cyprinus carpio*	Environmental exposure to PAHs and PCBs; Lab exposure to cyclophosphamide	Blood	0.5	2.5 M NaCI, 100 mM EDTA, 10 mM Tris, 10% DMSO, 1% Na sarcosinate	10	RT	2	nm	15 min	25 V (265–270 mA)	20 min (3°C; dark)	Pandrangi et al., 1995
*Psetta maximus*	Ethyl methane- sulphonate (EMS)	Blood, gill, liver and kidney	0.8	2.5 M NaCl, 100 mM Na EDTA, 10 mM Tris, 1% N-lauroylsarcosine, 1% Triton-X 100, 10% DMSO	nm	nm	1–2	>13	20–40 min	0.4–0.7 V/cm (300 mA)	10–20 min	Belpaeme et al., 1998
*Oncorhyncus mykiss, Ictalurus punctatus*	Aflatoxin B1	Blood, liver, kidney	0.75	2.5 M NaCl, 10 mM Tris-base, 0.1% sodium sarcosinate	10	RT	2	>13	15 min	20 V (300 mA)	25 min	Abd-Allah et al., 1999
*Pholis gunnellus*	Environmental exposure to PAHs and metals	Blood	1.0	2.5 M NaCl, 100 mM Na EDTA, 10 mM Tris, 1% Triton-X 100, 10% DMSO	10	4	1	12.3	30 min	25 V (300 mA)	15 min	Bombail et al., 2001
*Limanda limanda*	Environmental exposure to PAHs and PCBs	Blood	0.5	NaCl 2.5M, Na2 EDTA 0.1M, Tris base 0.01M, N-sarcosinate 1%, DMSO 10%, Triton X-100 1%	10	RT	1	13	15 min	23 V (390 mA)	20 min	Akcha et al., 2003
*Oreochromis niloticus*	Benzocaine	Blood	0.5	2.5M NaCl2, 100 mM Na2EDTA, 10 mM Tris, 1% sodium sarcosinate, 1% Triton X-100, 10% DMSO	10	4	1	>13	5–40 min	0.66 V/cm	5–40 min	de Miranda Cabral Gontijo et al., 2003
*Danio rerio*	4-nitroquinoline-1-oxide	Hepatocytes and gill cells	0.7	2.5M NaCl, 10 mM Tris, 100 mM EDTA, 1% Na-sarcosinate, 10% DMSO, 1% Triton X-100	10	nm	nm	nm	20 min	25 V (300 mA)	20 min	Diekmann et al., 2004b
*Petromyzon marinus*	Bisazir	Spermatozoa	nm	2.5M NaCl, 100 mM EDTA, 10 mM Tris base, 1% sodium lauryl sarcosinate, 1% Triton X-100, 1% DMSO	nm	4	1	>13	1 h	25 V (300 mM)	30 min	Ciereszko et al., 2005
*Geophagus brasiliensis, Cichla temensis, Hoplias malabaricus, Astyanax bimaculatus lacustres, Oreochromis niloticus, Cyprinus carpio, Steindachnerina insculpita*	Eutrophication	Blood	nm	NaCl 2.5 M; EDTA 100 mM; Tris 10 mM; N-laurolyl-sarcosine 1%; Triton-X 1%; DMSOn 10%	10	4	30 min	13	30 min	25 V (350 mM)	30 min (4°C; dark)	Grisólia et al., 2009
*Oncorhynchus mykiss*	Cryopreservation	Spermatozoa	nm	2.5 M NaCl, 100 mM Na2EDTA, 10 mM Tris, 1% Triton-X, 1% lauroyl sarcosine sodium salt, 4 mM lithium diiodosalicylate	8	4	1	12	20 min	25 V (300 mM)	10 min	Pérez-Cerezales et al., 2010
*Oncorhynchus mykiss, Danio rerio*	Trenbolone	Cell lines	0.7	100 mM EDTA, 2.5 M NaCl, 1% Triton X-100, 10% DMSO	13	4	1.5	nm	nm	25 V (310 mM)	nm	Boettcher et al., 2011
*Anguilla anguilla*	Glyphosate-based herbicide	Blood and liver	1.0	2.5 M NaCl, 0.1 M EDTA, 10 mM Tris, 1% Triton X-100	10	4	1	>13	20 min	25 V (300 mM)	15 min	Guilherme et al., 2012b
Ictalurus punctatus	Water and sediment from gypsum mining area	Ovary cell line	nm	2.5 M NaCl, 100 mM Na2EDTA, 10 mM Tris-HCl, 1% Na-sarcosinate, 1% Triton X-100, 10% DMSO	10	nm	nm	13	20 min	1 V/cm (300 mA)	20 min	Ternjej et al., 2013
*Catla catla*, *Labeo rohita*	Silver nanoparticles	Heart and gill cell lines	0.9	2.5 M NaCl, 100 mM EDTA, 10 mM Tris–HCl, 10% DMSO, 1% Triton X-100	10	4	1	>13	20 min	25 V (300 mM)	20 min	Taju et al., 2014

*nm, not mentioned; RT, room temperature*.

The Comet Assay adopted in different contexts has proved to be also valuable in the elucidation of the mechanisms of genotoxicity and DNA repair. In this direction, the implementation of a protocol with an extra step where nucleoids are incubated with DNA lesion-specific repair endonucleases has added greatly to the value of the Comet Assay (Azqueta and Collins, 2013), namely on the specific detection of oxidized bases and thus, identifying oxidative DNA damage as a harmful process underlying the genomic integrity loss. The use of endonuclease III (thymine glycol DNA glycosylase-Endo III) was initially proposed by Collins et al. (1993) to specifically target oxidized pyrimidines, while formamidopyrimidine DNA glycosylase (Fpg) was firstly adopted by Dusinska and Collins (1996) to signal oxidized purines. The adoption of this improved procedure in the field of environmental genotoxicology using piscine models took almost one decade, since, to the authors' knowledge, it was applied for the first time in 2003 (Akcha et al., 2003). This enzyme-modified assay has attracted particular attention in the last years, being applied either in whole organism (Tomasello et al., 2012), involving different tissues (blood, liver, and gill) (Aniagu et al., 2006), or cell line (Kienzler et al., 2012) testing. It was concluded that the scoring of the DNA damage encompassing oxidatively induced breaks increases sensitivity (Tomasello et al., 2012) and reduces the possibility of false negative results (Guilherme et al., 2012a when compared to the standard Comet Assay. This approach can be particularly informative when the additional breaks corresponding to net enzyme-sensitive sites are shown (Guilherme et al., 2012a). In the light of these positive outcomes, it seems clear that this specific tool has been underexploited.

Another technical development concerns the adoption of Comet Assay to evaluate the DNA repair ability of a specific tissue (Collins et al., 2001), namely through the *in vitro* assays for nucleotide excision repair (NER) and base excision repair (BER). For these assays, a DNA substrate containing specific lesions is incubated with an extract prepared from the tissue to test. The accumulation of breaks due to the incubation with that extract is a measure of DNA repair activity in the tissue (Azqueta et al., 2013). The few studies published using this type of assay include the detection of tissue-specificities of BER activity in *Xiphophorus* species, showing that brain possesses higher BER activity than gill and liver (Walter et al., 2001). The other available publications resulted from the work of the same research group and concern the application of BER (Kienzler et al., 2013a) and NER (Kienzler et al., 2013b) assays in fish cultured cells. Though the previous publications recommend the adoption of these DNA repair biomarkers as a complement the more classical genotoxicity endpoints (Kienzler et al., 2013a), their application has been clearly underestimated.

Blood has been, undoubtedly, the preferred tissue to perform Comet Assay in fish (e.g., Guilherme et al., 2010; Lourenço et al., 2010; Ternjej et al., 2010), mainly due to the easy sampling and availability of dissociated cells, a critical factor. All fish blood cells are nucleated which also represents an important practical advantage (comparing to mammals) for the assessment of genomic integrity. Nevertheless, other somatic tissues like liver, kidney and gills have been also frequently addressed (Guilherme et al., 2012b; Kumar et al., 2013; Velma and Tchounwou, 2013), as well as germ cells (Pérez-Cerezales et al., 2010). It is recognized that DNA strand breakage can be tissue- and cell-type-specific (Pandey et al., 2006). Hence, it is improbable that blood cells can reflect the type and extent of DNA damage occurring in other cell types. The choice of blood has been mainly determined by practical/technical reasons and rarely relied on the knowledge of a comparative performance with other target tissues. It has been stated that circulating cells are less sensitive, when compared to other types of cells (Frenzilli et al., 2009), but this is not a consensual assumption. As an example, a comparison between DNA damage in gill, kidney and blood tissues of *Therapon jarbua* following an exposure to mercuric chloride indicated the following order in terms of sensitivity: gill > kidney > blood cells (Nagarani et al., 2012). Guilherme et al. (2012b) stated that DNA damage in liver returned faster to the control level comparing to gills, which was regarded as an indication of a better adaptive behavior of hepatic cells, probably related with a higher capacity to maintain the genomic stability by detecting and repairing damaged DNA.

### Bivalves and other molluscs

Haemocytes are the most common target for genotoxicity assessment *in vivo* and *in vitro* in bivalves and gastropods (see Table 3). Although collection requires some skill, obtaining haemocytes from bivalve adductor muscles or haemocoel (e.g., pericardial) in bivalves and gastropods is proved to be feasible and able to yield cells apt for the Comet Assay in both number and quality. Still, it has been noted, concerning terrestrial snails, that broken or detached epiphragms may cause significant dehydration of tissues, hampering collection of haemolymph (Angeletti et al., 2013). Altogether, it is likely that haemolymph collection needs to be properly set and tested for each target organism. Gills have also been successfully employed since cell resuspension is easy enough to be assisted by gentle tissue splicing and “soft–pipetting” followed by low–speed centrifuging (≈2000 g) to remove debris and dead cells, without the need for treatment with collagenase (see Martins et al., 2012). Still, it has been shown that the baseline DNA strand breakage may greatly differ between organs.

**Table 3:** **Summary of the methodological developments on Bivalves and other Molluscs**.

**Experimental model**	**Contaminants tested**	**Cell type/tissue**	**Agarose (%)**	**Lysis buffer composition**	**Lysis conditions**			**Denaturation conditions**		**Electroforesis conditions**		**References**
					**pH**	**Temp. (°C)**	**Duration (h)**	**pH**	**Duration**	**Voltage**	**Duration**	
*Mytilus edulis; Ruditapes decussatus* (Bivalves)	Environmental exposure to PAHs, PCBs and metals;	Gills	1	Buffer A: 2.64% NaCl (w/v), 3.72% EDTA (w/v), 5 mM TRIS, 10% DMSO (v/v), 1% Triton-X 100	10	4	1	13	40 min	25 v	30 min	Martins et al., 2012, 2013
*Octopus vulgaris* (Cephalopods)	Heavy metal pollution	Gills, Digestive gland; “Kidneys”; Gonads	1	Buffer A	10	4	1	13	40 min	25 v	30 min	Raimundo et al., 2010
*Patella vulgata* (Gastropods)	Environmental exposure to PAHs	Haemolymph	1	n/m	10	5	1	13	45 min	25 V	30 min	Lewis et al., 2010
*Littorina littorea* (Gastropods)	Environmental exposure to OCP (organochlorine pesticides), PCBS, PAHs	Haemolymph	1	n/m	10	5	1	13	45 min	25 V	30 min	Noventa et al., 2011

*nm, not mentioned*.

The molluscan digestive gland, the analogous of the vertebrate liver and therefore of high relevance in toxicological studies, was shown to yield levels of single strand breakage likely too high (from autolytic processes) for a valid application of the Comet Assay without proper cell sorting and viability check (refer to Raimundo et al., 2010, in a study with the cephalopod *Octopus vulgaris* and Hartl et al., 2004 with the clam *Ruditapes philippinarum*). Recent advances have also shown the feasibility of obtaining adequate cultures of molluscan cells for *in vitro* studies using the Comet Assay (Michel and Vincent-Hubert, 2012) and even the possibility to cryopreserve mussel haemocytes (Kwok et al., 2013). Altogether, these advances certainly contribute to standardize the Comet Assay in biomonitoring and genotoxicity testing with bivalves and other molluscs.

### Terrestrial organisms

The Comet Assay in earthworms is performed on the small cells which constitute the most abundant class among the cellular population of the coelomic fluid, and that are the homologous, in worms, of vertebrate leucocytes. Cells are collected according to Eyambe et al. (1991), or by means of electric or ultrasonic stimulation. *Eisenia foetida* (*andrei*) is the most commonly used species, owing to the fact of being the one recommended by international guidelines for lethality and reproduction ecotoxicology studies; however, other species have been used, as for instance *A. caliginosa* (Klobučar et al., 2011), *Lumbricus terrestris*, *L. rubellus* (Spurgeon et al., 2003), *D. rubidus* and *M. benhami* (Fourie et al., 2007), among others (Vasseur and Bonnard, 2014).

Performing the Comet Assay in vegetal cells, however, present some particular difficulties (Gichner and Plewa, 1998). The rigid cellulose cell walls prevent DNA from leaving the cell, and are not easily eliminated with the usual alkaline treatment; so, nuclei isolation from tissues is necessary as a first step. However, the isolation procedure (either mechanical or chemical) may produce some degree of nuclear disruption, which could in some cases constitute a serious handicap. On the other hand, the high concentration of pigments and metabolites present in photosynthetic tissues (as leaves) tends to cause further damage to the isolated nuclei. To avoid this concern, root apical tissue is often preferred, but, in this case, the high rate of cell division may in turn be a problem. To reproducibly perform Comet Assay in leaves, some modifications to the standard procedure have been proposed, which includes a centrifugation through sucrose cushion, to eliminate disrupted nuclei and secure a higher fraction of undamaged nuclei (Peycheva et al., 2011). Recently, protocols have been developed to perform the Comet Assay in tree cell cultures from protoplasts following failure to obtain nude nuclei by the most common mechanical processes (Costa et al., 2012a) In spite of these difficulties, the Comet Assay has been successfully used in recent years to test the effects of Cr(VI) in *Pisum sativum* (Rodriguez et al., 2011), of Chlorfenvinphos and fenbuconazole in *Allium cepa* (Türkoğlu, 2012), cadmium-zinc (Cd-Zn) interactions in tobacco plant (Tkalec et al., 2014) or to demonstrate the correlation between the occurrence of B chromosomes and the DNA damage that is induced by the chemical mutagen, maleic hydrazide (MH), in *Crepis capillaris* plants (Kwasniewska and Mikolajczyk, 2014), among others. A recent revision (Ventura et al., 2013) is available.

There are a variety of working protocols of the comet assay for both birds and mammals (see Table 4). Circulating lymphocytes are used mainly as the test cell type because of its available and because it can be a non-invasive method of extracting sample (Azqueta and Collins, 2013). As described previously, the use of lesion-specific repair endonucleases has been employed in studies with in terrestrial organisms. This aspect brings to the Comet Assay a very interesting added value for targeting routes that are acting during exposure.

**Table 4:** **Summary of the methodological developments on Terrestrial organisms**.

**Experimental model**	**Cell type/tissue**	**Agarose (%)**	**Lysis buffer composition**	**Lysis conditions**			**Denaturation conditions**		**Electroforesis conditions**		**References**
				**pH**	**Temp. (°C)**	**Duration (h)**	**pH**	**Duration (min)**	**Voltage**	**Duration**	
*Aporrectodea caliginosa; Amynthas diffringens; Dendrodrilus rubidus; Eisenia fetida; Microchaetus benhami* (earthworms)	Coelomocytes	0.5	Buffer A: 2.5 M NaCl, 100 mM EDTA, 10 mM Tris, 10% DMSO, 0.2 M NaOH, 1% Triton X-100	10	4°C	15	13	20 min	35 V (300 mA)	10 min	Fourie et al., 2007
*Eisenia fetida; Eisenia sp*. (earthworms)	Coelomocytes	n/m	Buffer B:2.5 M NaCI, 100 mM EDTA, 10 mM Tris-HCl, 10% DMSO, 1% triton X-100	10	4°C	24	13	5 min	25 V (300 mA)	5 min (4°C)	Espinosa-Reyes et al., 2010
*Aporrectodea caliginosa; Eisenia fetida* (earthworms)	Coelomocytes	0.8	Buffer B	10	4°C	1	13	15 min	35 V (300 mA)	20 min (4°C)	Klobučar et al., 2011
*Eisenia andrei Bouché* (earthworms)	Coelomocytes	1.0	Buffer C: 2.5 M NaCI, 100 mM EDTA, 10 mM Tris, 10% DMSO, 1% triton X-100, 0,2 NaOH	10	4°C	Overnight	13	25 min (RT)	0.74 V/cm	30 min (RT)	Vernile et al., 2013
*Eisenia fetida* (earthworms)	Coelomocytes	n/m	Buffer B	10	4°C	1, 5	n/m	20 min (RT)	25 V (300 mA)	20 min	Saez et al., 2014
*Ciconia ciconia; Milvus migrans* (birds)	Blood	0.7	Buffer D: 2.5 M NaCl, 10 mM Na_2_EDTA, 10 mM Tris, 10% DMSO, 1% sodim sarcosinate, 1% Triton X-100	10	4°C	1	12.8	20 min (4°C)	1.6 V/cm (300 mA)	20 min (4°C)	Pastor et al., 2004
*Hirundo rustica* (birds)	Blood	1.0	Buffer E: Lysis Solution, Trevigen, 10% DMSO	10	4°C	1	12.1	45 min	25 V (250 mA)	10 min	Bonisoli-Alquati et al., 2010
*Ctenomys torquatus* (rodents)	Blood	0.75	Buffer B	10		1–2 weeks	12.6	31 min	25 V (300 mA)	30 min	da Silva et al., 2000a,b
*Apodemus sylvaticus* (rodents)	Blood	0.7	Buffer C	10	n/m	n/m	13	20 min	25 V (300 mA)	15 min	Festa et al., 2003
*Microtus pennsylvanicus* (rodents)	Blood	0.75	Buffer F: 2.5 M NaCl, 10 mM Na2EDTA, 10 mM Tris Base, 1% n-lauryl sarcosinate, 1% Triton X-100	10	4°C	Overnight	13.1	30 min	27 V	20 min	Knopper et al., 2005
*Rattus rattus; Mus musculus* (rodents)	Blood	n/m	Buffer B	10	4°C	2 weeks	13	30 min (4°C)	25 V (300 mA)	30 min	León et al., 2007
*Mus spretus* (rodents)	Blood	0.7	Buffer C	12	4°C	1	12.8	20 min	1 V/cm	20 min	Mateos et al., 2008
*Apodemus sylvaticus* (rodents)	Blood	0.5	Buffer B	10	4°C	1	n/m	15 min	0.7 V/cm (300 mA)	10 min	Lourenço et al., 2013
*Nicotiana tabacum* (plants)	Leaves	0.75	Buffer C	10		1	13	20 min	25 V (300 mA)	20 min (4°C)	Gichner and Plewa, 1998
*Allium cepa; Nicotiana tabacum* (plants)	Roots, leaves	n/m	n/a	n/a	n/a	n/a	13	15 min	26 V (300 mA)	20 min (4°C)	Ghosh et al., 2010
*Allium cepa* (plants)	Roots	n/m	n/a	n/a	n/a	n/a	13	12 min	0.75 V/cm (300 mA)	15 min (4°C)	Panda et al., 2011
*Pisum sativum* L (plants)	Leaves	1.4	n/a^*^	n/a^*^	n/a^*^	n/a^*^	12.6	30 min (4°C)	0.45 V/cm	10 min	Peycheva et al., 2011
*Pisum sativum* L (plants)	Roots, leaves	1	n/a	n/a	n/a	n/a	13	15 min	0.74 V/cm	15 min	Rodriguez et al., 2011
*Crepis capillaris* (plants)	Leaves	1	n/a	n/a	n/a	n/a	13	15 min	15 V (340 mA)	15 min (4°C)	Kwasniewska and Mikolajczyk, 2014
*Allium cepa* (plants)	Roots	0.75	n/a	n/a	n/a	n/a	13	12 min	0.75 V/cm (300 mA)	15 min (4°C)	Pakrashi et al., 2014
*Nicotiana tabacum* L (plants)	Seeds, roots, leaves	1	n/a	n/a	n/a	n/a	13	10 min	0.8 V/cm (300 mA)	20 min	Tkalec et al., 2014
*Picea abies (plants)*	*Protoplasts from embryogenic cultures*	1	Buffer B	10	4	1.5	13	40	25 V	30 min	Costa et al., 2012a

*n/m, not mentioned; n/a, not applicable (For isolation of nuclei, tissues are treated with 400 mM Tris buffer pH 7.5, and then finely and gently sliced with a razor blade)*;

* *nuclei isolation was performed by: (1) cutting of the tissue, (2) homogenizing in phosphate buffered saline (PBS), (3) adding protease inhibitor phenylmethylsulphonyl fluoride (PMSF), (4) centrifuging and (5) re-suspending in PBS*.

## Correlations with other biomarkers

### Amphibians

The combination of Comet Assay, to detect DNA strand breaks, with the evaluation of other biomarkers to determine the effects of contaminants in exposed organisms has been performed in many studies. Some of those studies show a positive correlation between the results given by the Comet Assay and other biomarkers. For instance, in the studies performed by Mouchet et al. (2005a,b, 2006a,b), a positive correlation between DNA strand breaks detection and micronucleus induction was observed most of the times. This result was expected since the Comet Assay measures primary DNA damages and the micronucleus test reflects irreparable lesions that result from the non-repaired or inappropriately repaired primary DNA damages, which are likely to be inherited by subsequent generations of cells. In another study Liu et al. (2006) investigated the role of reactive oxygen species (ROS) in the herbicide acetochlor-induced DNA damage on *Strauchbufo raddei* tadpole liver and the results showed a positive correlation between DNA damage and malondialdehyde (MDA) formation and a negative correlation between DNA damage and total antioxidant capability. This result showed that the herbicide acetochlor induce DNA damage through the formation of ROS. Zhang et al. (2012) conducted a study to evaluate cadmium-induced oxidative stress and apoptosis in the testis of frog *Fejervarya limnocharis*, which also showed a positive correlation between DNA damage, lipid peroxides and ROS formation and gluthatione determination, showing the role of oxidative stress to damage DNA of these cells. These studies show the importance of the inclusion of the Comet Assay in a battery of tests that contribute to determine the chain of events leading to the effects observed and to determine the type of damages to DNA.

### Piscine models

As a sign of maturity, in the last years a particular attention has been devoted to the interference of non-contamination related factors (biotic and abiotic) with the genotoxicity expression. This is a critical knowledge to allow a correct assessment of the contribution of chemical contamination to the DNA damage measured. In this direction, hypoxia, and hyperoxia, known as important stressors in the aquatic environment, were tested in *Cyprinus carpio*, revealing that both conditions increase oxidative DNA damage (approximately 25% compared to normoxic conditions) (Mustafa et al., 2011). Another study demonstrated that acute extreme exercise results in oxidative DNA damage in *Leuciscus cephalus*, suggesting that fish living in fast flowing and polluted waters are at increased risk (Aniagu et al., 2006). The effects of age, gender, and sampling period were also investigated (Akcha et al., 2004). In adult fish (*Limanda limanda*), DNA breaks were higher in males than in females, whereas the opposite trend was observed for juveniles. Regardless of gender, the extent of DNA damage was higher in the adult comparing to juvenile fish. It was also suggested that the formation of DNA lesions can be modulated by seasonal variables, namely those related to variations in lipid content, biotransformation activity and/or to spawning cycles (Akcha et al., 2004). It was hypothesized that anesthesia used before tissue sampling can have confounding influences on the DNA integrity evaluation. Still, Nile tilapia exposed to benzocaine showed that this anesthetic does not affect Comet Assay results (de Miranda Cabral Gontijo et al., 2003).

The assumption that the Comet Assay can be successfully applied to monitor effects of environmental disturbances emerged unanimously from the majority of fish studies using this technique (e.g., Ciereszko et al., 2005; Srut et al., 2010). Tough a more skeptical perspective can detect in this unanimity a self-worth and self-legitimation positioning, it is also clear that it represents a strengthening of the goodness of the assertion. It has been suggested that the ecotoxicological consequences of a genomic instability and its correlation with DNA breaks measured by the Comet Assay deserves a special attention (Jha, 2008). To gain ecological relevance, a mechanistic association between genotoxic stress and effects at higher biological levels should be identified, contributing to predict deleterious effects mainly at population level (e.g., abundance and reproduction impairments). The controversy whether adverse effects of anthropogenic genotoxicants can be associated to the decline of fish populations has been the leitmotiv for some recent studies. A complete life-cycle test was carried out with zebrafish (*Danio rerio*) and the model genotoxicant (4-nitroquinoline-1-oxide) seeking for a causal linkage between genotoxic effects and ecotoxicological risk (Diekmann et al., 2004a,b). It was observed a reduction of egg production, which would have led to fish extinction according to a mathematical simulation (Diekmann et al., 2004a), concomitantly with DNA damage induction (Diekmann et al., 2004b). However, this study failed on demonstrating a direct evidence that genotoxicity is functionally related to reduced egg production (Diekmann et al., 2004a). The assessment of the consequences of germ cell DNA damage on progeny outcomes has been regarded as a strategy to signal potential long-term effects of aquatic genotoxicants in fish, since genetic damage in such cells, if unrepaired or misrepaired, can be passed on to future generations (Devaux et al., 2011). In this direction, it was demonstrated a positive correlation between the DNA damage in sperm from parental fish (*Salmo trutta* and *Salvelinus alpinus*) exposed to the alkylating genotoxicant model methyl methanesulfonate and the incidence of skeletal abnormalities in the offspring, clearly suggesting that DNA damage had been inherited (Devaux et al., 2011). In a subsequent study, spermatozoa of *Gasterosteus aculeatus* were exposed *ex vivo* to MMS before *in vitro* fertilization and a relationship between abnormal embryo development in the progeny and sperm DNA damage was demonstrated (Santos et al., 2013). It was also revealed that sperm of *Oncorhynchus mykiss* maintains its ability to fertilize in spite of having DNA damage, although embryo survival was affected (Pérez-Cerezales et al., 2010). The risk evaluation of the impact of DNA-damaged germ cells in the reproduction is particularly relevant in animals with external fertilization/embryo development (Pérez-Cerezales et al., 2010), like fish, since both gametes and embryos can be directly exposed to waterborne genotoxicants. This approach can represent an additional contribution to predict the impact of DNA damage on recruitment rate, progeny fitness, and thereby, on the population dynamics. A recent multi-generation study with zebrafish (*D. rerio*) involving a chronic exposure to MMS demonstrated impairments in survival, growth, reproductive capacities and DNA integrity (Faßbender and Braunbeck, 2013). Furthermore, due to the transfer of mutations and inherited DNA damage to the next generation, the offspring was subject to elevated teratogenicity and mortality, pointing out a causal relationship between genotoxicity and the decline of wild populations (Faßbender and Braunbeck, 2013).

### Bivalves and other molluscs

It must be noted that there are many reports showing reduced genotoxic effects of organic toxicants to molluscs through studies *ex situ* (Parolini and Binelli, 2012; Martins et al., 2013), which, nonetheless, does not relate with technical constraints of the Comet Assay (at least the standard protocols for the alkaline assay are proven to be perfectly effective) but rather on the mechanisms underneath the bioactivation of organic toxicants by multi–function oxidases that, in vertebrates, are responsible for the production of ROS and genotoxic metabolites (Peters et al., 2002). Nevertheless, studies *in situ* with bivalves, at least, often yield good agreement between Comet Assay data and background levels of mixed toxicants, especially organic (Pereira et al., 2011; Martins et al., 2012; Michel et al., 2013). Still, some authors noted the influence of environmental confounding factors, especially, season–related, highlighting increased oxidative stress and DNA strand breaks during warmer months (Almeida et al., 2011; Michel et al., 2013).

The enzyme–modified Comet Assay to detect oxidative DNA damage is just starting to be applied to molluscs, in an attempt to understand the mechanisms underlying DNA damage in these organisms, a subject that still remains largely unknown. It is the case, for instance, of the work by Dallas et al. (2013), who failed to detect Ni–driven Fpg–sensitive (oxidative) DNA damage in the haemocytes of tested mussels, which contradicts *in vitro* studies with humans cells (refer to Cavallo et al., 2003). In another example, Michel and Vincent-Hubert (2012) disclosed that hOGG–1 is more effective in the detection of oxidative damage than alkylated sites (even compared to Fpg) in *D. polymorpha* gill cells exposed *in vitro* and *in vivo* to a known genotoxicant such as B[α]P. These apparent contradictions showed just how much little is known about the causes and mechanisms of DNA damage and repair in molluscs. In fact, Comet Assay data often yields contradictory or non-linear relations when contrasted to bioaccumulation of genotoxicants and biomarkers related to oxidative stress (such as lipid peroxidation or the activity of antioxidant enzymes), depending on substance, species, and conditions of assessment (e.g., Noventa et al., 2011; Martins et al., 2013). This, again, calls for the need to break way toward the understanding of the fundamental mechanisms underlying genotoxicity in molluscs and their differences to vertebrates, for which most genotoxicity assessment approaches have been devised.

### Terrestrial organisms

*E. fetida* is extensively used as a compost worm because of its potential to degrade wastes, and has been reared in farms and laboratories for decades. Its continuous exposure to toxic compounds, especially those deriving from agricultural practice, may have been an evolutionary factor for the species. The selective appearance of specific metabolic ways for the detoxification of certain compounds may also result in the activation of other genotoxicants, as has been shown in other species (*Mus musculus* compared with *Apodemus silvatycus*, Acosta et al., 2004). On the other hand, and by a similar reasoning, worms which are native of polluted areas may have developed resistance to those compounds present in their environment.

## Discussion and future prespectives

The Comet Assay presents several significant advantages over other commonly used assays for genotoxicity studies. Its applicability to both eukaryotic and prokaryotic organism and its use in almost any cell type makes this assay a test very verifiable, reliability, relatively rapidly in data collection and realistic correlation are characteristics also provided by this technique. However, one of the virtues of this assay is unquestionably its cost-effectiveness, compared to many other techniques.

The discussion about the importance of inter-specific differences in sensibility, and on the meaningfulness of using substitute instead of native or target species, is long-lived and still alive, and concerns the core of the toxicological thinking. Indeed, extrapolation is the Achilles heel of toxicology, hence the particular attention given to protocol enhancement and standardization, albeit the need to reason that each case study and each organism need their own set of technical specifications and interpretation requirements, especially considering non-model and moreover, native, species.

There is a wide variety of internal procedures of laboratories where the Comet assay is carried out. As underlined in a previous review article (Frenzilli et al., 2009), the development of suitable guidelines for standardizing Comet Assay protocols is imperative to achieve a harmonization and inter-laboratory calibration. This is also a critical issue to the generalized recognition of Comet Assay as environmental monitoring tool and to allow its integration in regulatory genotoxicological studies. It should be required to the scientist community and to the regulatory agencies to make a meta-analysis or a simple comparison of results obtained from the literature.

Although the Comet Assay has been applied in studies of amphibians, for instance, since the late 1990s, a standardized method to perform the assay and to measure and report this effect does not exist. This represents a disadvantage that limits the comparison with other studies. Despite that, the use of Comet Assay in these organisms is increasing, although it is still limited to the detection of DNA damage. This shows that there is a great potential for development and application of this technique to ecotoxicological studies and environmental risk assessments using amphibians as bioindicator species. The elucidation of the type of DNA damage that is generated and the accurate monitoring of DNA repair through lesion-specific enzymes during the Comet Assay protocol, will add value to this assay in future ecotoxicological studies for exposure assessment and effects on these organisms. Additionally, it could also help to determine the potential causes of their decline in specific environments.

Despite the evidence here highlighted toward a functional association between genotoxicity measured at individual level and a negative impact at population level, so far, DNA damage detected by Comet Assay in fish (as well as in other animal models) has failed to garner sufficient recognition to be incorporated into national and international risk assessment protocols, even though the comparison between this and other potential biomarkers as already showed higher efficiency in the distinction between impacted and reference sites (Costa et al., 2012b). The unequivocal and convincing (mainly for public regulatory agencies) demonstration of its ecological relevance is probably the greatest challenge to Comet Assay on the next decade (goal extensible to majority of biomarkers currently adopted in environmental toxicology).

Another of the many technical constraints that need to be circumvented before the Comet Assay can be efficiently and profusely applied to a wider range of organisms relate to the collection and nature of samples *per se*. For instance, one of the major problems in ecotoxicity terrestrial testing is the high amount of product needed to perform the Comet Assay test. In the case of earthworms, a possible method to reduce the amount of test material required is to inject the test solution directly in the coelomic cavity of the earthworms; this is how was conducted the recently reported Comet Assay study of functionalized-quantum dots (QDs) and cadmium chloride on *Hediste diversicolor* and *E. fetida* coelomocytes. Results demonstrated that functionalized-QDs (QDNs) and cadmium chloride induced DNA damages through different mechanisms that depended on the nano- or ionic nature of Cd (Saez et al., 2014). Spiked soil should be allowed to stabilize for a sufficient period before starting the exposition test to performing the Comet Assay. This time, necessary to reach a status of equilibrium similar to that established in natural conditions, is probably too short in most studies. On the other hand, the nature and circumstances of soil in the real polluted areas may dramatically affect the bioavailability of xenobiotics. Time and exposure to the action of weather tends to have a homeostatic effect, decreasing the access of toxicants to the internal medium of living organisms. This partially accounts for the surprisingly mild effects frequently observed in areas which chemical analysis have shown to be heavily polluted (Alexander and Alexander, 2000; Borràs and Nadal, 2004; Vasseur and Bonnard, 2014). As a consequence, experiments with spiked soil could tend to show a higher degree of toxic effects, being more sensitive but also, possibly, less realistic. Still regarding this issue, a way to avoid the large amounts of sample needed in a conventional growth test in soil consists in treating only the exposed root tips. For example, *Allium cepa* root tips were treated with TiO_2_ nanoparticles dispersions at four different concentrations (12.5, 25, 50, 100 mg/mL). The bio-uptake of TiO_2_ in particulate form was the key cause of ROS generation, which in turn was probably the cause of the DNA aberrations and genotoxicity (Ghosh et al., 2010; Panda et al., 2011; Pakrashi et al., 2014).

Overall, these few examples clearly illustrate that the application of the Comet Assay in ecogenotoxicity assessment remains as purposeful as challenging. The swift integration of novel methodological improvements to the protocol with this field of research, such as DNA repair enzyme modifications, shows that ecotoxicologists are constantly improving approaches and protocols. Furthermore, it must be noticed, as hereby demonstrated, that ecotoxicology is probably one of the most diversified and complex field of research where genotoxicity assessment is surveyed as routine. As such, one may expect another further decades of successful, although constantly improving, application of this versatile protocol.

## Internet resources

Frost, D. R. (2011). Amphibian Species of the World: An Online Reference, v. 5.5. Electronic Database accessible at http://research.amnh.org/vz/herpetology/amphibia/. American Museum of Natural History, New York, USA.

### Conflict of interest statement

The authors declare that the research was conducted in the absence of any commercial or financial relationships that could be construed as a potential conflict of interest.
